# Effects of vagotomy on cardiovascular and heart rate variability alterations following chronic normobaric hypoxia in adult rabbits

**DOI:** 10.1186/s40659-018-0207-2

**Published:** 2018-12-20

**Authors:** Julio Alcayaga, Rodrigo Del Rio, Esteban A. Moya, Matías Freire, Rodrigo Iturriaga

**Affiliations:** 1 Laboratorio de Fisiología Celular, Facultad de Ciencias, Universidad de Chile, Santiago, Chile; 2 Laboratorio de Control Cardiorrespiratorio, Departamento de Fisiología, Pontificia Universidad Católica de Chile, Santiago, Chile; 3 Centro de Envejecimiento y Regeneración (CARE-UC), Pontificia Universidad Católica de Chile, Santiago, Chile; 4 Centro de Excelencia en Biomedicina de Magallanes (CEBIMA), Universidad de Magallanes, Punta Arenas, Chile; 5 Laboratorio de Neurobiología, Facultad de Ciencias Biológicas, Pontificia Universidad Católica de Chile, Santiago, Chile; 6 Present Address: Precision Medicine, Pfizer, Santiago, Chile; 7 Present Address: Division of Physiology, Department of Medicine, UCSD, San Diego, USA

**Keywords:** Heart rate variability, Chronic normobaric hypoxia, Cardiovascular regulation, Sympathetic control, Vagal activity, Poincaré plot, Spectral components

## Abstract

**Background:**

chronic hypoxia increases basal ventilation and pulmonary vascular resistance, with variable changes in arterial blood pressure and heart rate, but it’s impact on heart rate variability and autonomic regulation have been less well examined. We studied changes in arterial blood pressure, heart rate and heart rate variability (HRV) in rabbits subjected to chronic normobaric hypoxia (CNH; PB ~ 719 mmHg; F_I_O_2_ ~ 9.2%) for 14 days and assess the effect of autonomic control by acute bilateral vagal denervation.

**Results:**

exposure to CNH stalled animal weight gain and increased the hematocrit, without affecting heart rate or arterial blood pressure. Nevertheless, Poincaré plots of the electrocardiographic R–R intervals showed a reduced distribution parallel to the line of identity, which interpreted as reduced long-term HRV. In the frequency domain, CNH reduced the very-low- (< 0.2 Hz) and high-frequency components (> 0.8 Hz) of the R–R spectrograms and produced a prominent component in the low-frequency component (0.2–0.5 Hz) of the power spectrum. In control and CNH exposed rabbits, bilateral vagotomy had no apparent effect on the short- and long-term HRV in the Poincaré plots. However, bilateral vagotomy differentially affected higher-frequency components (> 0.8 Hz); reducing it in control animals without modifying it in CNH-exposed rabbits.

**Conclusions:**

These results suggest that CNH exposure shifts the autonomic balance of heart rate towards a sympathetic predominance without modifying resting heart rate or arterial blood pressure.

## Background

Hypoxic challenges induce physiological homeostatic responses that reduce the mismatch between oxygen supply and tissue demand. Peripheral and central chemoreceptors are responsible of generating systemic reflex ventilatory and cardiovascular adjustments to cope with a hypoxic challenge [1–3], although hypoxia is mainly sensed by the peripheral chemoreceptors (carotid and aortic bodies). In response to acute hypoxia, ventilation increases in frequency and/or volume, which in turn increased PaO_2_, reduced PaCO_2_ and increased pH, thus increasing oxygen gradient and tissue supply [3, 4]. Concomitantly, an increase in systemic arterial blood pressure and heart rate augments the oxygen delivery rate to the tissues, aiding to restore the normal oxygen supply. If the hypoxic challenge is maintained, as occurs in high altitude exposure, there is an increase in resting ventilation [5] and heart rate [6], and the development of pulmonary hypertension [7, 8]. However, systemic arterial blood pressure has been reported to remain unchanged in chronic hypobaric hypoxia [7, 9] or increased in chronic normobaric hypoxia [8, 10, 11]. Thus, cardiovascular response during long-term hypoxia appear to be modified by barometric pressure that may modify the physiologic responses to hypoxia [12–14]. It is generally accepted that the hypoxia-induced changes in cardiovascular parameters may occur in tandem with an increase in the sympathetic drive [15]. Heart rate variability (HRV) indexes in the time and frequency domains have been widely used in humans and animals to estimate the autonomic balance to the heart rate without any invasive intervention [16]. Spectral analysis of HRV has two major oscillatory components defined as: (1) a low-frequency (LF) band, related to the combined sympathetic and parasympathetic influences, and (2) a high-frequency (HF) band, related to vagal influences and respiratory sinus arrhythmia [16–18]. Spectral analysis has shown that chronic sustained hypobaric hypoxia modifies the LF and HF bands of HRV in humans [19] and experimental animals [20–22], even in the absence of systemic hypertension. Moreover, cats and rats exposed to chronic intermittent normobaric hypoxia, a condition that mimics the obstructive sleep apnea syndrome, showed early shifts in LF and HF components of the HRV that preceded the development of systemic hypertension [17, 18, 23]. Therefore, HRV analysis estimates autonomic changes following exposure to hypoxia. Despite the studies showing HRV alterations in response to hypobaric hypoxia less is known about the effect of chronic sustained normobaric hypoxia on HRV. Moreover, different physiological responses to the same low PO_2_ level [12, 14] or in response to exercise [24, 25] have been described in hypobaric versus normobaric hypoxic conditions, suggesting that hypoxia and hypobaria could evoke different physiological responses. Therefore, we studied the effects of chronic hypoxia (O_2_ inspiratory fraction (F_I_O_2_) ~ 9.2%) on autonomic control of heart rate in rabbits maintained in normobaric conditions (PB ~ 719 mmHg) for 2 weeks and assessed the balance in autonomic regulation of HRV with the elimination of vagal parasympathetic control after acute bilateral vagotomy.

## Methods

### Animals

Experiments were performed in male New Zealand White rabbits, 11 control and 8 exposed to chronic normobaric hypoxia (CNH) for 14 days. After recording the cardiovascular variables in baseline normoxic conditions both vagus were cut, in 6 control and 6 CHN rabbits to assess contribution of the parasympathetic tone on physiological parameters. Experiments were conducted in accordance to the guidelines of the National Fund for Scientific and Technological Research (FONDECYT, Chile) and the Guide for the Care and Use of Laboratory Animals (National Research Council of the National Academies, USA). Bio-Ethics Committees from the Facultad de Ciencias of the Universidad de Chile, and Facultad de Ciencias Biológicas of the Pontificia Universidad Católica de Chile approved the experimental protocols.

### Chronic normobaric hypoxia exposure

**Fig. 1:**
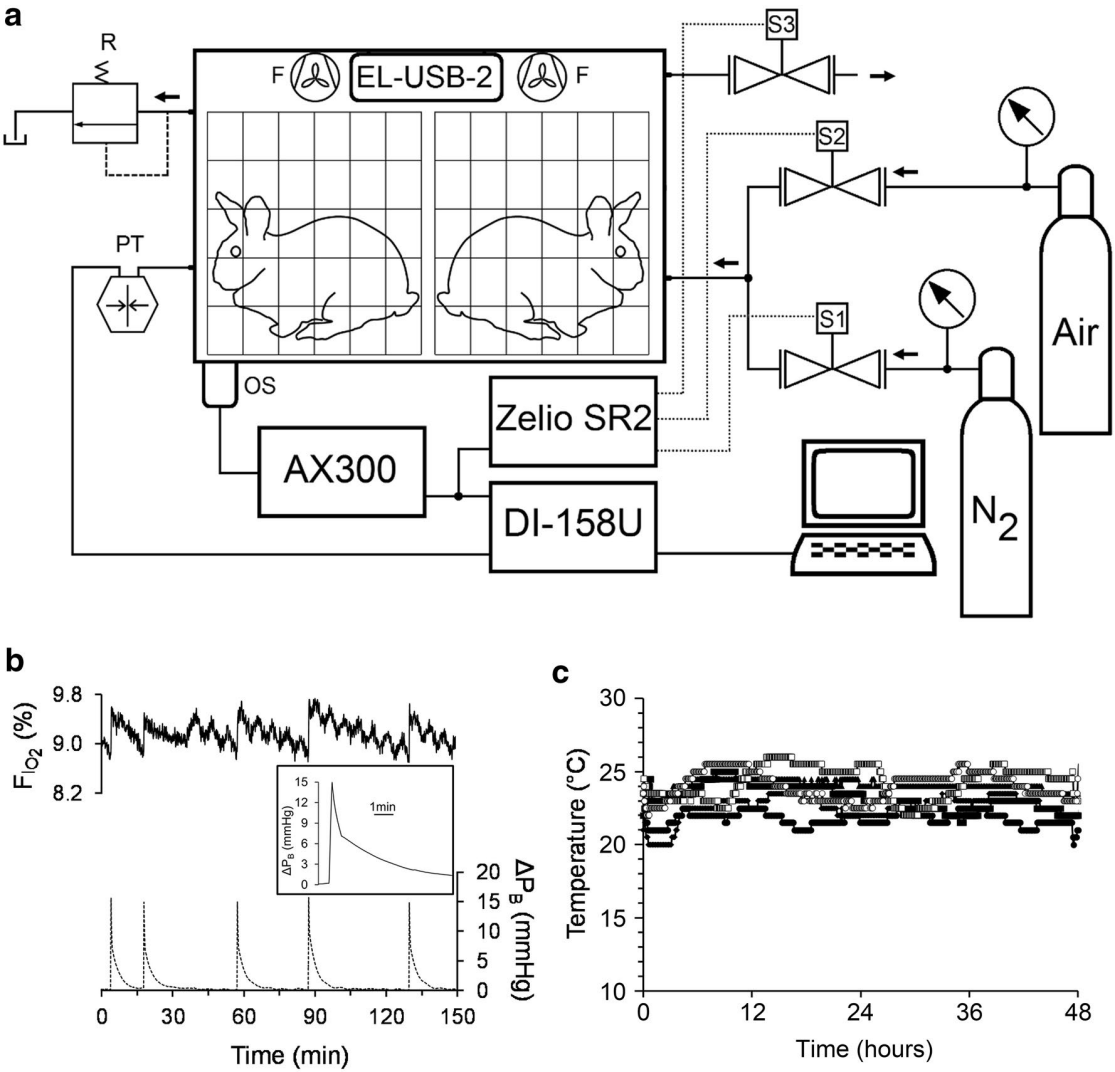
Experimental chamber and environmental measures in the chamber during hypoxic conditioning. **a** The chamber could contain two rabbits caged independently. Chamber O_2_ level (F_I_O_2_) was monitored continuously with an O_2_ sensor (OS). If F_I_O_2_ values were > 10% or < 8.9%, then the O_2_ sensor triggered a relay controller to activate a solenoid valve to admit either N_2_ (S1) or air (S2), respectively, and another (S3) to exhaust chamber gas (arrows on the gas lines indicate direction of gas flow). A safety release valve (R) opened if the chamber pressure increased to 15 mmHg ≥ atmospheric pressure. Chamber gas was mixed continually by fans (F, n = 4). **b** Every time the F_I_O_2_ reached about 8.7% (upper panel) the system injected compressed air, increasing F_I_O_2_ up to around 9.8% (upper panel) and the pressure in the chamber near 15 mmHg (lower panel). After the initial pressure increase, the pressure fell (inset) with an initial fast exponential decay due to the exhaust valve and a second slower decay due to pressure leak through the lid of the chamber. **c** Temperature recordings of 48 h periods in different experiments (n = 7). No temporal pattern of temperature variation was observed in the chamber

In each experiment, two rabbits were simultaneously exposed to normobaric hypoxia as previously described [26] (Fig. 1a), with a total of eight rabbits exposed to CNH. Briefly, individually caged (D × H × W; 55 cm × 42 cm × 38 cm) rabbits were placed in a 300 L (60 cm × 50 cm × 100 cm) Lucite^®^ chamber. The oxygen content (F_I_O_2_) was monitored continuously by an oxygen sensor (AX300, Teledyne Analytical Instruments, USA) whose output was connected to a programmable automatic controller (Zelio SR2 B121BD, Schneider Electric, France) that activated solenoid valves (2026BV172, Jefferson Solenoid Valves, USA), admitting N_2_ or compressed air to the chamber if F_I_O_2_ values were over ~ 9.8% or below ~ 8.7%, respectively; a relief output valve opened simultaneously with the input valves and remained opened for 40 s after the closure of the input valves. Additionally, mechanic relief valves opened whenever pressure inside the chamber exceeded 17 mmHg above the ambient pressure. Thus, the mean F_I_O_2_ was maintained near 9.2%. The atmosphere of the chamber was mixed by 4 fans. CO_2_ produced by the ventilation of the rabbits was trapped using CaCO_3_ (250 g) and the urinary ammonia with H_3_BO_3_ (60 g). The chamber was opened every other day for approximately 5 min to clean the cages, to replenish the food and water and to replace CaCO_3_ and H_3_BO_3_ when necessary. The pressure inside the chamber was measured continuously with a gauge transducer (Statham P20). Throughout the conditioning period (14 d), chamber pressure and F_I_O_2_ signals were recorded by an analog-to-digital acquisition system sampling at 1 Hz (DI-158U, DATAQ Instruments Inc., USA), and the chamber’s temperature was recorded at 5-min intervals by a data logger (EL-USB-2, Lascar Electronics Inc., USA). Reference barometric pressure values for Santiago (717.2 ± 0.3 mmHg; altitude: 567 m) and relative humidity (63.3 ± 2.5%) were obtained daily from the web database (http://164.77.222.61/climatologia/) of the Dirección Meteorológica de Chile of the Dirección General de Aeronáutica Civil.

The CNH chamber design and control system, F_I_O_2_, pressure and temperature dynamics over a 24 h period are display in Fig. 1. At the beginning of each hypoxic exposure, flushing the chamber for 4 min with N_2_ decreased the F_I_O_2_ from 21.11 ± 0.04% (8 experiments) to 9.87 ± 0.30%, with a time constant of 2.15 ± 0.05 min (n = 8), reaching a stable value of 9.14 ± 0.08% after one additional min. When the F_I_O_2_ level fell below ~ 8.7% (Fig. 1b), the system automatically injected compressed air in the chamber for 20 s, thus maintaining a mean F_I_O_2_ value of 9.18 ± 0.03% (14 periods of 24 h). The admission of air (or N_2_ when F_I_O_2_ increased over ~ 9.8%) induced a small rapid increase in the pressure inside the chamber (Fig. 1b) with a mean maximal value of 14.7 ± 0.1 mmHg (n = 980 admissions in 13 periods of 24 h), which returned to the baseline level within 10 min (Fig. 1b). The mean interval between consecutive air admissions to the chamber was 16.3 ± 1.4 min (10 periods of 48 h), with a maximal mean interval of 43.8 ± 2.2 min (10 periods of 48 h). Because of the admission of compressed gases into the chamber, the mean pressure increased in 1.3 ± 0.1 mmHg (13 periods of 24 h) within the chamber, resulting in a mean pressure of 718.5 ± 0.4 mm Hg (13 periods of 24 h) during the hypoxic challenge. On the other hand, the mean temperature within the chamber remained constant (23.0 ± 0.2 °C; 14 periods of 24 h; Fig. 1c), presenting periodic variations in a single experiment but with no constant pattern throughout the different experiments (Fig. 1c).

### Physiological recordings

Rabbits were anesthetized initially with ketamine/xylazine (75/7.5 mg/kg, i.m.) and thereafter maintained with sodium pentobarbitone (6–9 mg/kg every 30 min, i.v.). The rabbits exposed to CNH (n = 8) were recorded within 5 h after their removal from the hypoxic chamber between 8:30 am and 01:00 pm, while control animals (n = 11) were recorded at 8:30 am. The rabbits were placed in supine position on a heated pad. We cannulated their trachea and connected the cannula to a heated tachograph which output was connected to a differential pressure transducer. The right saphenous vein and the left femoral artery were cannulated for drug application (i.e. sodium pentobarbitone) and arterial blood pressure measurement, respectively. The arterial cannula was connected to a low volume pressure transducer. The electrocardiogram (ECG) was recorded using the II lead. Three stainless steel 22G-needle electrodes were localized in the insertion of the right (G1) and left (GND) front legs, and in the left (G2) rear leg. The signals from the pressure transducer (pulse pressure) and the ECG were sampled at 2 kHz and recorded by our data acquisition system (PowerLab8, ADInstruments, Australia). Systolic, diastolic, pulse and mean blood pressure and heart rate were analyzed offline using the blood pressure module of data analysis software (LabChart Pro, ADInstruments, Australia). After recording the cardiovascular variables in baseline normoxic conditions, bilateral transection of both vagus nerves rostral to the nodose ganglia were performed in a subset of control (n = 6) and CNH (n = 6) rabbits chosen randomly to analyze the contribution of the parasympathetic tone on the measured parameters.

### Heart rate variability

The ECG were recorded for 10 min in each animal and the R–R intervals series were exported from LabChart Pro as text file and processed using Kubios Software (v1.1 SP1) from the Biomedical Signal Analysis Group, University of Kuopio, Finland [27]. Time domain analysis of the HRV was performed using Poincaré plots. Briefly, the standard deviation of the (R–R)_n_ intervals respect to the next (R–R)_n+1_ interval was computed. Short-term variability was defined by the points above the contingency line (SD1) and the long-term variability correspond to the points along the contingency line (SD2). Power spectral analysis was used to calculate HRV in the frequency domain. Briefly, the R–R time series was detrended using a smoothness prior algorithm, with α = 800 and an interpolation rate of 8 Hz. The spectral bandwidth was 0–2.1 Hz, with a spectral resolution of 0.1 Hz, using a window width of 420 points with no overlap. The spectral analysis was performed over the whole spectrum without assuming specific ranges within the bandwidth. Spectral analysis of HRV has two major oscillatory components defined as a low-frequency (LF) band, thought to be related to the combine sympathetic and parasympathetic influences, and a high-frequency (HF) band related to vagal influences and the respiratory sinus arrhythmia [16–18]. Thus, the LF-to-HF ratio (LF/HF) of HRV is used as an indirect index of cardiac sympathovagal balance.

The frequency bands of R–R intervals spectra were defined according to the power spectra obtained from control rabbits: (i) very-low frequency (VLF), DC-0.2 Hz, (ii) low frequency (LF), 0.2–0.5 Hz and (iii) high frequency (HF): 0.5–2.1 Hz. Calculations considered the relative power of the LF band and the HF band normalized to the total spectral power (standardized units, S.U.); the LF/HF was calculated from these values.

### Blood glucose levels

Venous blood was withdrawn using a Teflon catheter from the ear marginal vein, and blood glucose level was measured using commercial strips and a glucose meter (Accu-Chek^®^ Active, Roche, Germany).

### Statistical analysis

All data is presented as mean ± SEM. Statistical analysis of data was performed with GraphPad Prism (version 6.07 for Windows, GraphPad Software, San Diego California, USA). Non-normally distributed variables: body weight, hematocrit, SD1, SD2, LF, HF, and LF/HF ratio. Normally distributed variables: blood glucose, heart rate, cardiac cycle duration, cardiac contraction time, systolic pressure, diastolic pressure, HRV total power. Mean arterial pressure as well as HRV power bands were normally distributed before but not after vagotomy. Comparison between two groups was performed using parametric (Student´s t-tests) or nonparametric (Mann–Whitney or Wilcoxon test) tests, according to the data structure. Comparison between more than two groups was performed using nonparametric analysis of variance (Kruskal–Wallis) test. Statistical decision level was set at p < 0.05.

## Results

### CNH effects on body weight, blood glucose and hematocrit

The mean body weight of control animals (1.87 ± 0.08 kg; n = 11) was like the initial mean weight of animals exposed to CNH (1.79 ± 0.10 kg; n = 8). After 14 days, the body weight of control rabbits increased significantly to 2.27 ± 0.10 kg (p < 0.05). In contrast, the average body weight of CNH exposed rabbits was not significantly modified after 2-wks of CNH exposure (1.79 ± 0.10 kg vs. 1.78 ± 0.11 kg). Moreover, at the end of the CNH exposure rabbits had lower body weights than predicted values based on unexposed rabbits (2.27 ± 0.10 kg, p < 0.01).

CNH affected hematocrit but did not alter blood glucose levels. The mean hematocrit increased from 40.3 ± 0.7% at the beginning of the CNH period to 55.8 ± 0.9% after 14 days of CNH (p < 0.001). The mean blood glucose levels, measured after anesthetic induction and prior to physiological recordings, were similar in control and CNH rabbits (7.99 ± 0.51 mmol/L, n = 5 and 8.55 ± 0.47 mmol/L, n = 5, respectively; p > 0.05).

### Effects of CNH on resting cardiovascular variables in basal conditions

Table 1 contains the mean values for the cardiovascular variables for control and CNH rabbits measured during a 10-min recording of consecutive heart beats after the induction of anesthesia. Rabbits exposed to 14 days of CNH had no statistically significant differences (p > 0.05) in arterial pressure, heart rate, cardiac cycle duration and contraction time, compared to control rabbits. Specifically, heart rate and mean arterial as well as systolic and diastolic arterial pressures were slightly but not significantly increased in CNH rabbits compared to the values in control rabbits (Table 1). Similarly, arterial pulse pressure was slightly but not significantly reduced in CNH rabbits with respect to control animals (Table 1).

**Table 1:** Cardiovascular variables in control rabbits and in rabbits exposed to chronic normobaric hypoxia (CNH) for 14 days under basal conditions

Group	Systolic pressure (mmHg)	Diastolic pressure (mmHg)	Mean arterial pressure (mmHg)	Pulse pressure (mmHg)	Heart rate (BPM)	Cycle duration (ms)	Time to peak (ms)
Control							
x̄ ± SEM	74.6 ± 4.4	54.5 ± 3.9	61.7 ± 3.8	20.1 ± 1.5	229.6 ± 14.8	270.0 ± 15.0	82.9 ± 6.0
n	11	11	11	11	11	11	11
CNH							
x̄ ± SEM	78.9 ± 6.0	62.1 ± 5.0	68.8 ± 5.3	16.8 ± 1.4	252.4 ± 21.8	250.0 ± 22.0	80.6 ± 2.6
n	8	8	8	8	8	8	8

### Cardiovascular parameters after bilateral vagotomy

**Fig. 2:**
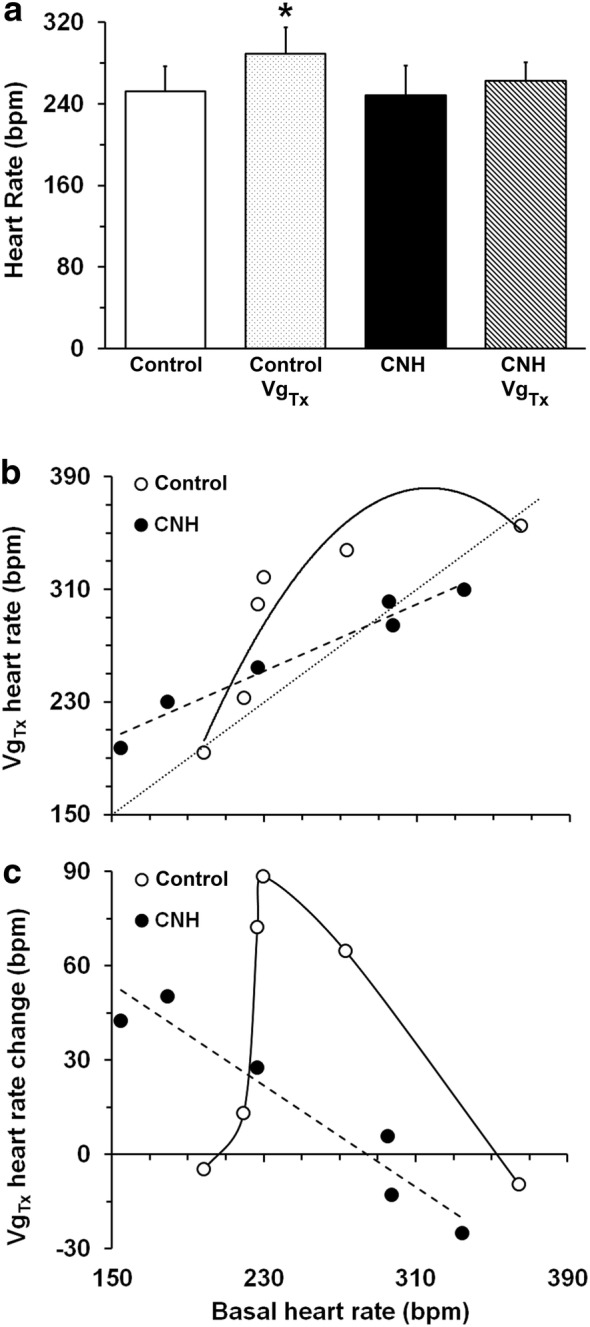
Heart rate before and after bilateral vagotomy in control and CNH rabbits. **a** Mean heart rate was significantly increased in control (n = 6) but not in CNH rabbits (n = 6) after bilateral vagotomy (Vg_Tx_). **b** Scatter plot of mean heart rate after bilateral vagotomy as a function of basal mean heart rate. Dotted line: line of identity. **c** In control rabbits, changes in mean heart rate were maximal in the midrange of basal heart rate; in CNH rabbits, changes were inversely and linearly related to basal mean heart rate. Control rabbits: empty circles, continuous line. CNH rabbits: filled circles, segmented line. Bars: SEM. *p < 0.05

Most cardiovascular variables were affected by bilateral vagotomy (Table 2) in control rabbits. After vagotomy, mean heart rate increased significantly from 252.10 ± 24.58 bpm to 281.00 ± 24.35 bpm (p < 0.05; Fig. 2a). Similarly, after vagotomy, mean systolic, diastolic, and pulse arterial pressure increased (p < 0.05), and mean arterial blood pressure also increased (p < 0.05). Conversely, bilateral vagotomy did not modify significantly these cardiovascular variables in the CNH rabbits (Table 2).

**Table 2:** Effects of bilateral vagotomy (Vg_Tx_) on cardiovascular variables in control and rabbits exposed to chronic normobaric hypoxia (CNH)

Group	Systolic pressure (mmHg)	Diastolic pressure (mmHg)	Mean arterial pressure (mmHg)	Pulse pressure (mmHg)	Heart rate (BPM)
Control					
x̄ ± SEM	79.0 ± 7.3	57.0 ± 6.6	64.3 ± 5.7	22.0 ± 2.8	252.1 ± 24.6
Control + Vg_Tx_					
x̄ ± SEM	93.7 ± 6.0*	68.1 ± 5. 9*	75.4 ± 5.1*	25.7 ± 3.4*	281.0 ± 24.4*
n	5	5	6	5	6
CNH					
x̄ ± SEM	86.0 ± 6.8	63.5 ± 4.6	72.7 ± 5.4	22.5 ± 3.6	248.1 ± 29.5
CNH + Vg_Tx_					
x̄ ± SEM	91.9 ± 10.0	67.7 ± 6.9	76.5 ± 7.9	24.2 ± 3.4	262.8 ± 17.9
n	6	6	6	6	6

Differences induced by vagotomy within the same group; * p < 0.05

Changes in heart rate after bilateral vagotomy depended on the resting heart rate. In control rabbits, heart rate was almost unaffected at the extremes of the pre-vagotomy range (low, 194 or high, 364 bpm) but was increased maximally near midrange (Fig. 2b, c). In contrast, changes in heart rate in CNH rabbits were linearly related to the heart rate prior to vagotomy (Fig. 2b, c).

### Heart rate variability (HRV): time-domain analysis

#### Analysis in intact conditions

**Fig. 3:**
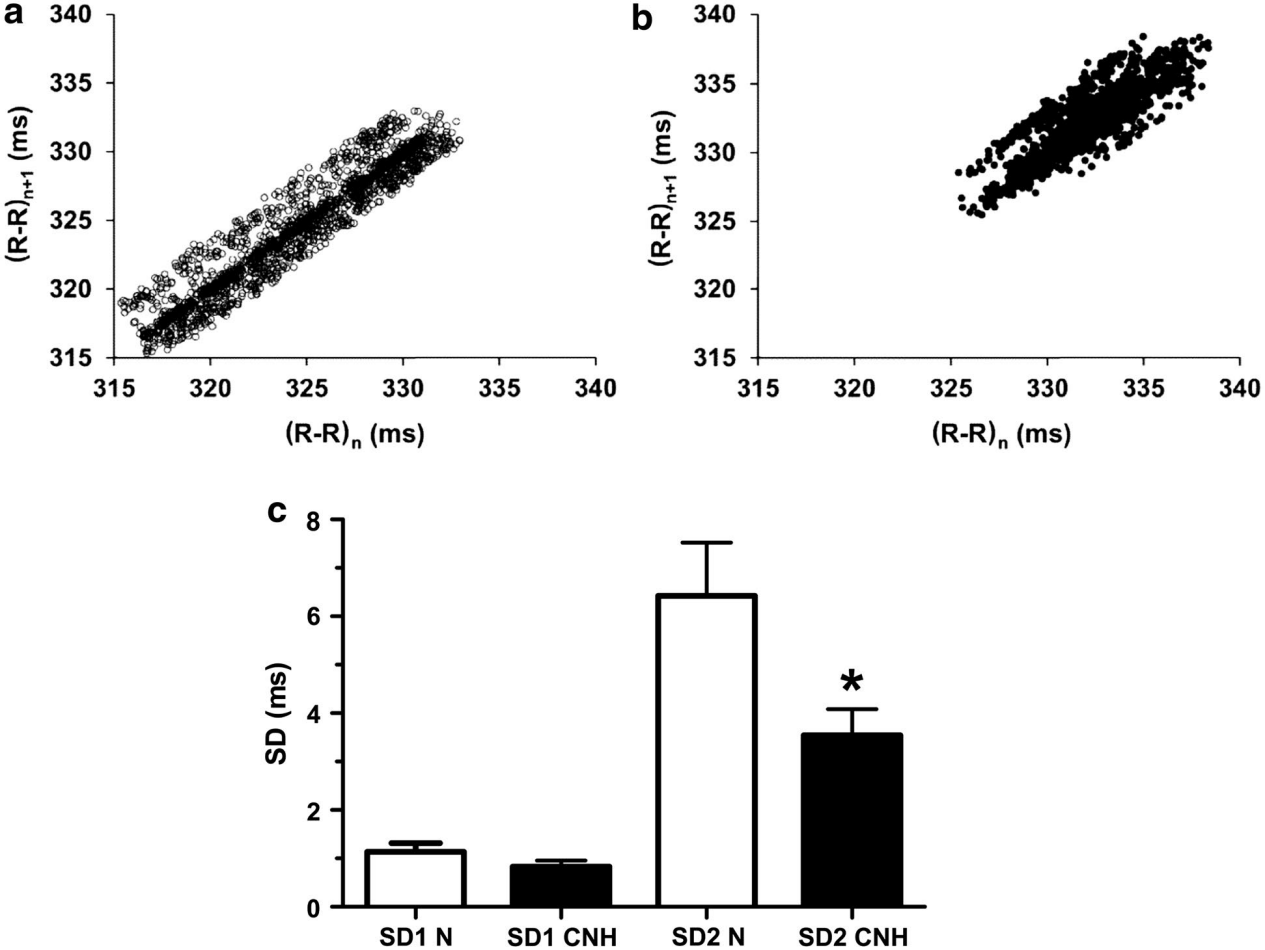
Effect of chronic normobaric hypoxia on time-domain analysis of heart rate variability. **a** Relationship between the duration of a cardiac cycle (RR interval, n; (R–R)_n_) and that of the next one (RR interval, n + 1; (R–R)_n+1_) and in a control rabbit. **b** Relationship between the duration of nth RR interval, (R–R)_n_, and the following RR interval, (R–R)_n+1_, in a CNH rabbit. **c** Mean SD1 was similar in control and CNH rabbits, while mean SD2 was significantly lower in CNH than in control rabbits. Empty bars: control rabbits (n = 11). Filled bars: CNH rabbits (n = 8). *p < 0.05. Dispersion bars: SEM

Poincaré plots of the R–R interval series were constructed and the short-term (SD1; minor axis) and long-term (SD2; major axis) HRV variabilities were calculated. Chronic normobaric exposure did not modify the short-term, but reduced the long-term variability (Fig. 3a, b). Control rabbits had a SD1 mean value of 1.14 ± 0.18 ms and rabbits exposed to CNH had a mean SD1 value of 0.83 ± 0.13 ms (p > 0.05, Fig. 3c). On the other hand, CNH rabbits showed a 1.8-fold reduction in SD2 compared to control rabbits (3.54 ± 0.54 ms versus 6.42 ± 1.1 ms; p < 0.05, Fig. 3c).

#### Analysis after acute bilateral vagotomy

**Fig. 4:**
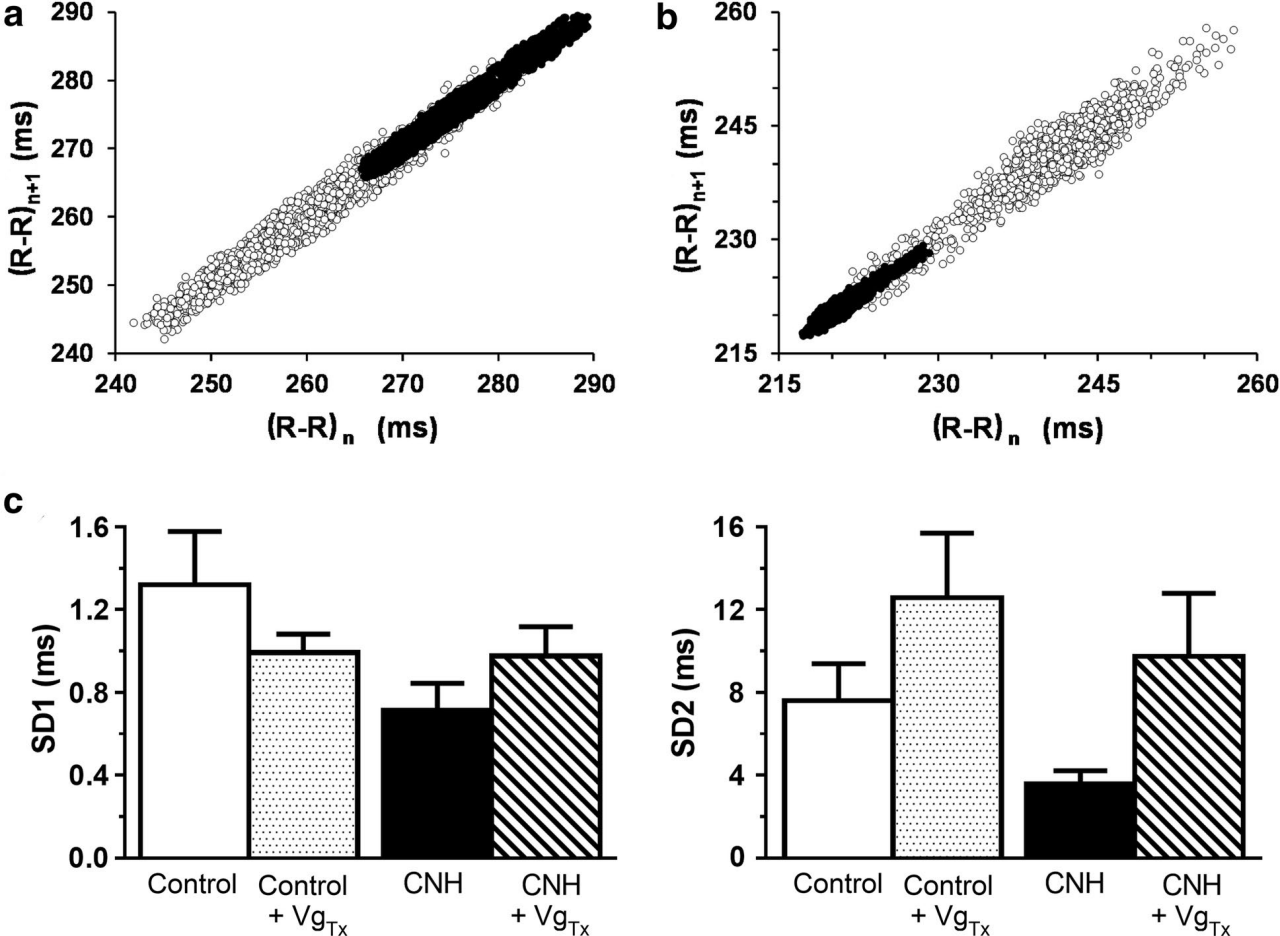
Effect of vagotomy (Vg_Tx_) in the time-domain analysis of heart rate variability. **a** Relationship between the duration of the following cardiac cycle ((R–R)_n+1_) and the previous one ((R–R)_n_) in a control animal, before and after vagotomy. **b** Relationship between the duration of (R–R)_n+1_ and (R–R)_n_ in a CNH rabbit, before and after vagotomy. **c** Mean SD1 (left) and SD2 (right) from control (n = 6) and CNH (n = 6) rabbits before and after vagotomy (Vg_Tx_). Mean SD1 and SD2 were statistically unaffected by vagotomy. Before vagotomy: filled circles. After vagotomy: empty circles. Empty bars: control rabbits. Dotted bars: control rabbits after vagotomy. Filled bars: CNH rabbits. Lined bars: CNH rabbits after vagotomy. Dispersion bars: SEM

The effects of bilateral vagotomy on the time-domain analysis of HRV are illustrated in Fig. 4. Vagotomy in control (Fig. 4a) and CNH rabbit (Fig. 4b) had small effects on short-term (SD1), and long-term (SD2) HRV. In control rabbits, the mean value of SD1 was not modified by vagotomy, with an initial average of 1.32 ± 0.26 ms before and 0.99 ± 0.09 ms after denervation (p > 0.05, Fig. 4c). Control rabbits had mean SD2 values of 7.59 ± 1.80 ms before and 12.58 ± 3.12 ms after vagotomy (p > 0.05, Fig. 4c). Similarly, CNH rabbits had mean SD1 values of 0.72 ± 0.32 ms before and 0.98 ± 0.34 ms after bilateral vagotomy (p > 0.05, Fig. 4c). The mean values of SD2 in CNH rabbits were 3.57 ± 1.60 ms before and 9.75 ± 7.43 ms (p > 0.05, Fig. 4c). Thus, acute bilateral vagotomy did not modify HRV in the time-domain for both control and CNH-exposed rabbits.

### Heart rate variability (HRV): frequency domain analysis

#### Analysis in intact conditions

**Fig. 5:**
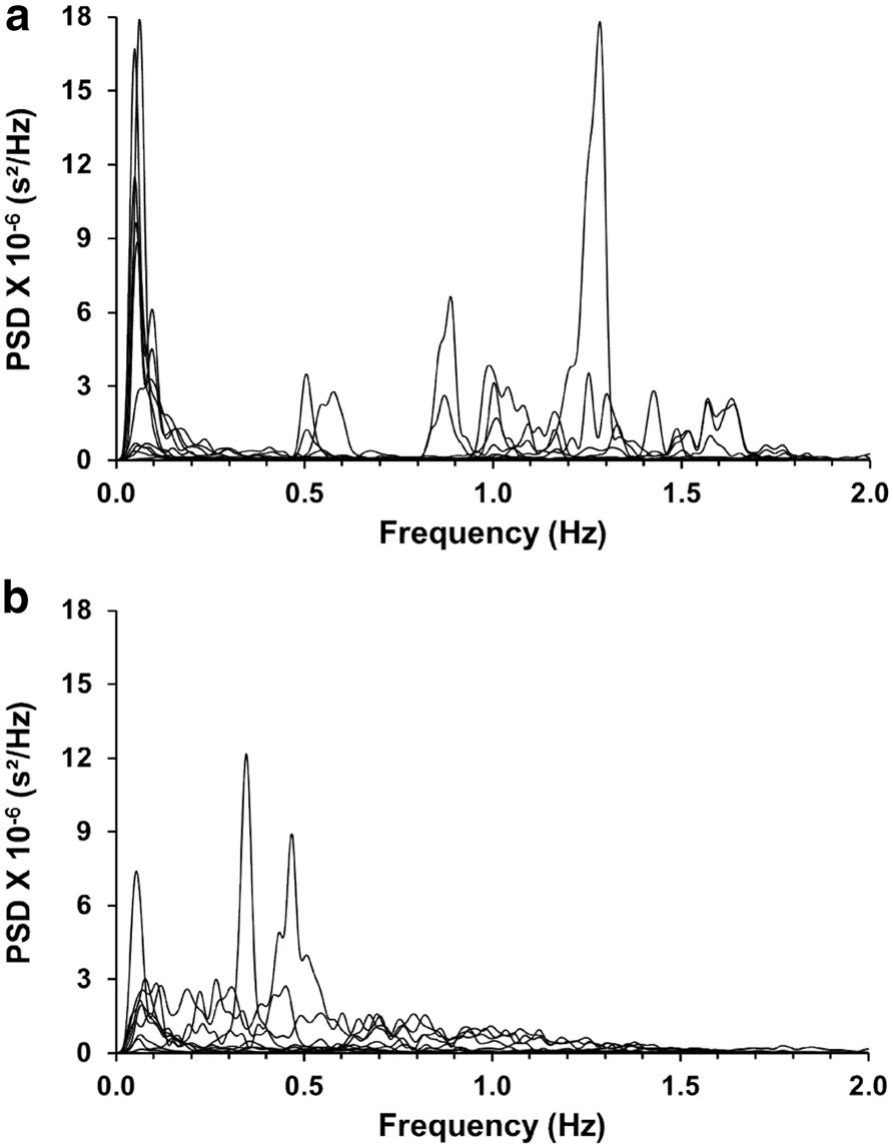
Power spectra of the R–R intervals from ten consecutive minutes of ECG recording. **a** Power spectra of control rabbits (n = 11) had components with the greatest power at the very-low-frequency (< 0.2 Hz) and high frequency (0.5–2.1 Hz) bands, with additional components with lower power at low-frequency (0.2–0.5 Hz) band. **b** Power spectra of CNH rabbits (n = 8) had reduced power at very-low-frequency band, almost no components at the high-frequency band but an increase in the power of components between in the low-frequency band. *PSD* power spectral density

**Fig. 6:**
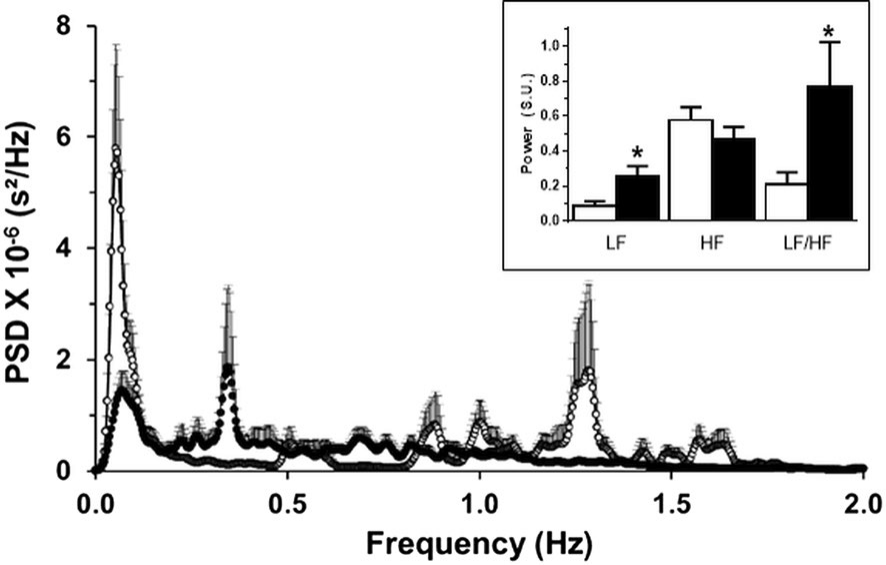
Mean power spectra of the R–R intervals from ten consecutive minutes of ECG recording. The mean power spectrum of CNH rabbits (dotted line, filled circles; n = 8) showed a large reduction of the components at the very low (< 0.2 Hz) and high (> 0.8 Hz) frequency bands, with respect to the mean power spectrum of control rabbits (continuous line, empty circles; n = 11). The low-frequency band between 0.2 and 0.5 Hz was increased in CNH rabbits with respect to the mean power spectrum of control rabbits. Inset: mean values, in standardized units (S.U.), of low frequency (LF), high frequency (HF) and LF/HF ratio in control (empty bars) and CNH rabbits (filled bars). *Significantly different from control rabbits; p < 0.05. Dispersion bars: SEM. *PSD* power spectral density

Individual spectrograms of the variation in RR Intervals of each of the control and CNH rabbits are displayed in Fig. 5. We found that the spectra of control rabbits consistently had maximal power peaks in the very-low (< 0.2 Hz) and high- (0.8–1.9 Hz) frequency bands (Fig. 5a). Spectrograms from rabbits exposed to CNH showed a marked flattening of their power spectrum and a reduction of frequency components below 0.2 Hz and above 0.8 Hz (Fig. 5b), compared to spectra obtained from control rabbits (Fig. 5a). However, HRV total power was similar in control and CNH rabbits (220.8 ± 45.2 ms^2^ vs. 162.7 ± 35.0 ms^2^, control vs. CNH, respectively; p > 0.05). Total power was differentially disturbed in control and CNH-conditioned rabbits. Compared to control rabbits, the mean spectrogram of CNH rabbits had decreases (p < 0.01) in the power of the very low-frequency band (< 0.2 Hz, Fig. 6) and in the high frequency band (> 0.8 Hz, Fig. 6), and a concomitant increase (p < 0.01) in the power of the low frequency range (0.2–0.5 Hz, Fig. 6). Accordingly, the LF/HF ratio was greater in rabbits exposed to CNH compared to control rabbits (0.77 ± 0.26, 0.21 ± 0.07, respectively; p < 0.05). Indeed, rabbits exposed to CNH had a ~ 3.5-fold increase in the LF/HF ratio compared to control rabbits (Fig. 6).

#### Analysis after acute bilateral vagotomy

**Fig. 7:**
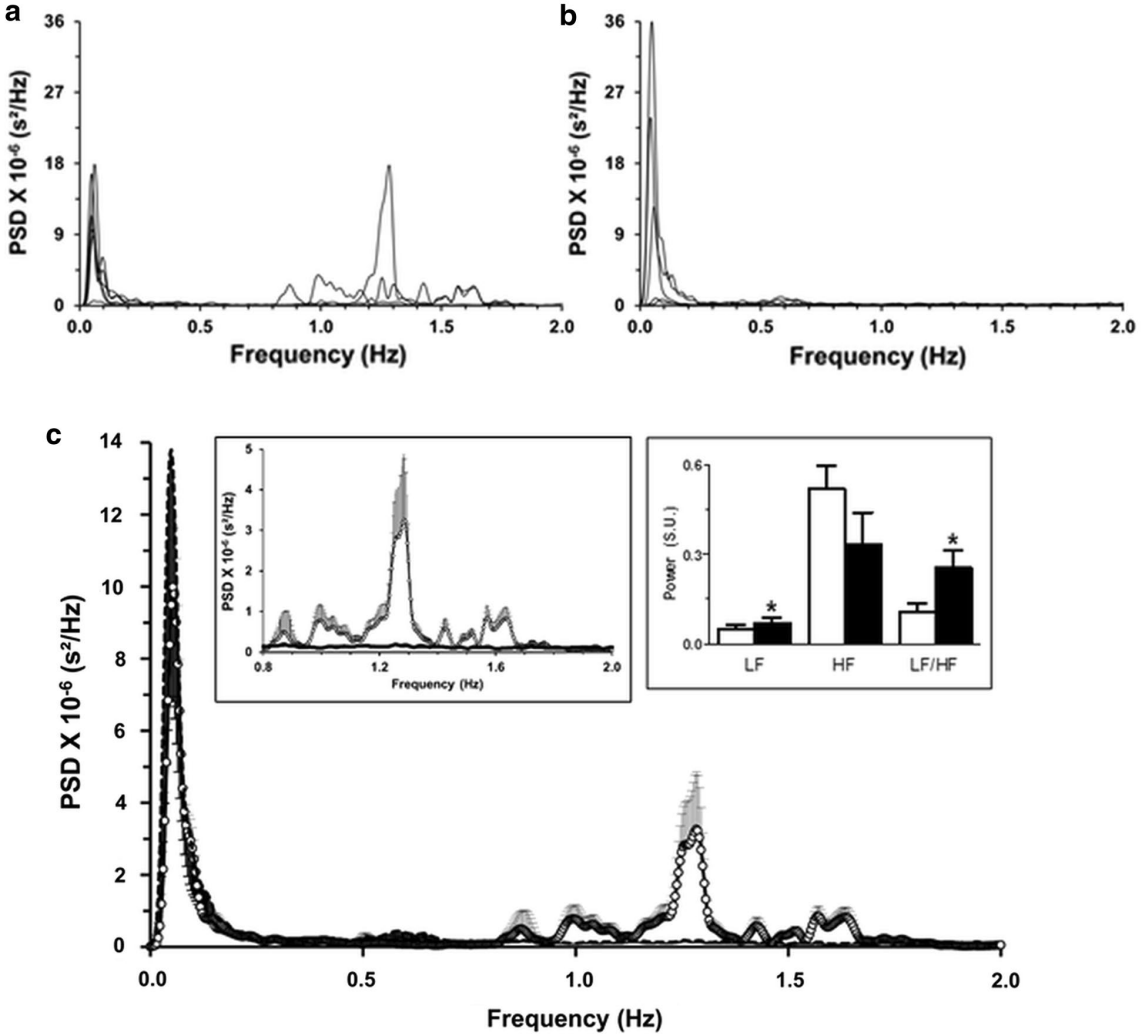
Power spectra of the R–R intervals of control rabbits (n = 6) before and after bilateral supra-nodose vagotomy. **a** Before vagotomy, the prominent components of the power spectral density (PSD) occurred in the very-low- (< 0.2 Hz) and high- (0.8–1.7 Hz) frequency bands. **b** After vagotomy, the power of the very-low-frequency components (< 0.2 Hz) increased, while the high-frequency band decreased. **c** The mean power spectrum of vagotomized rabbits (filled circles, continuous line) increased in the very-low frequency band compared to that before vagotomy (empty circles), while the power in the high frequency band decreased, nearly absent between 0.8 and 1.7 Hz compared to that before vagotomy. Left inset: mean power spectra of the high-frequency band (0.8–2.0 Hz). Right inset: mean values, in standardized units (S.U.), of low frequency (LF), high frequency (HF) and LF/HF ratio in control conditions (empty bars) and after bilateral vagotomy (filled bars). *Significantly larger than intact condition; p < 0.05. Dispersion bars: SEM. *PSD* power spectral density

In control rabbits, the total power of the spectra remained comparable before and after vagotomy (319.2 ± 72.7 ms^2^ vs. 272.0 ± 96.8 ms^2^, control vs. CNH, respectively; p > 0.05). However, the total power was redistributed. Compared to their pre-vagotomy spectrograms (Fig. 7a), bilateral vagotomy: (i) decreased power in the high-frequency band, especially > 0.7 Hz; (ii) increased power slightly in the low frequency band, 0.2–0.5 Hz (Fig. 7b), and (iii) increased power in the very-low-frequency band, < 0.2 Hz (p < 0.01, Fig. 7c). Acute vagotomy did not affect the mean low-frequency peak (0.056 ± 0.002 Hz before and 0.055 ± 0.004 Hz after vagotomy; p > 0.05, Fig. 7c). The mean high-frequency peak decreased from 1.33 ± 0.17 Hz in the intact condition to 0.94 ± 0.22 Hz after denervation (p > 0.05). Thus, the LF band and the LF/HF ratio increased from 0.05 ± 0.01 and 0.11 ± 0.03 to before to 0.07 ± 0.02 and 0.25 ± 0.06 respectively, after bilateral vagotomy (p < 0.05, Fig. 7c).

**Fig. 8:**
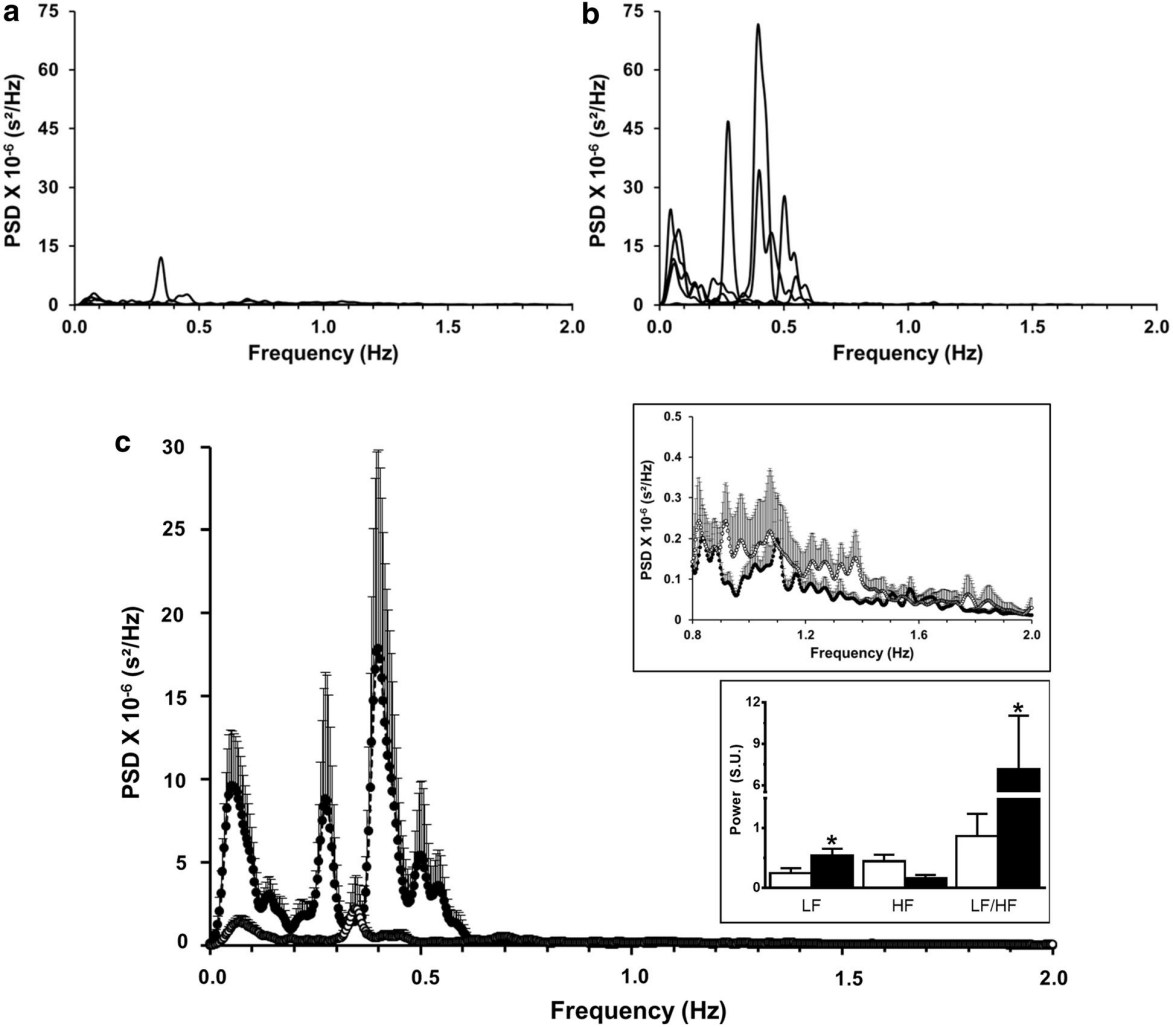
Power spectra of the R–R intervals of CNH rabbits (n = 6) before and after bilateral supra-nodose vagotomy. **a** The principal components of the power spectra of CNH rabbits before vagotomy occurred in very-low- and low- frequency bands (< 0.5 Hz) and almost no components at high frequency band (0.5–2.1 Hz). **b** After vagotomy, the power very-low- and low- frequency bands (< 0.6 Hz) increased. **c** The mean power spectrum of vagotomized animals (filled circles) showed a large increase in the power of the lower frequency bands compared to those before vagotomy (continuous line, empty circles), while the components in the 0.8 and 1.7 Hz range remained weak and appeared unaffected. Upper inset: mean power spectra of the 0.8–2.0 Hz frequency range. Lower inset: mean values, in standardized units (S.U.), of low frequency (LF), high frequency (HF) and LF/HF ratio in control conditions (empty bars) and after bilateral vagotomy (filled bars). *Significantly different from than intact condition; p < 0.05. Dispersion bars: SEM. PSD: power spectral density

In CNH rabbits, comparing their spectra pre and post vagotomy (Fig. 8), the total power of the spectra increased due to increases in power at very-low- and low- frequency bands (< 0.6 Hz). Average total power of the spectra increased after bilateral vagotomy from 134.7 ± 35.3 ms^2^ to 742.3 ± 210.9 ms^2^ (p < 0.05). The mean power spectrum increased in peak amplitude of the very-low-frequency band (0.07 ± 0.005 Hz vs. 0.8 ± 0.21 Hz, pre vs. post vagotomy, respectively). Two additional peaks were evident in the low frequency band at 0.3 and 0.4 Hz (Fig. 8b, c). Nevertheless, the power content of the high frequency band (0.6–2.0 Hz) remained small and unaffected by vagotomy (0.39 ± 0.16 ms^2^ vs. 0.28 ± 0.05 ms^2^, pre vs. post vagotomy, respectively; p > 0.05; Fig. 8c). Moreover, the LF band and the LF/HF ratio increased from 0.25 ± 0.08 and 0.87 ± 0.37 before vagotomy to 0.54 ± 0.12 and 7.18 ± 3 after vagotomy (p < 0.05; Fig. 8c).

## Discussion

The main findings of this study are that exposure of rabbits to CNH for 2 weeks: (i) prevented weight gain, (ii) decreased long-term variability of the HRV, (iii) shifted the spectral distribution of the HRV from the HF band towards LF band suggesting a reduction in parasympathetic modulation on HRV despite the absence of changes in arterial blood pressure and heart rate. Together, these results suggest that CNH induced autonomic deregulation characterized by sympathetic/parasympathetic imbalance.

We found that body weight did not increase significantly during the 14 days of CNH exposure. However, growth curves for control rabbits indicate that the animals should have increased their weight around 20% after 14 days [27, 28]. A slight decrease in weight has been observed in mice [29], rats [30] and piglets [31] under a similar CNH paradigm, with a fast recovery of weight and weight gain after 7 and 14 days, respectively, after returning to normoxia [27]. Thus, the CNH exposure appears not to impair permanently the metabolism of adult rabbits as it does to adult rats exposed to postnatal hypobaric hypoxia [32]. Stagnation of body weight and pulmonary hypertension has been associated with acute and chronic hypoxia [7, 33]. Pulmonary arterial pressure increases with hypoxia [7], but only hypobaric hypoxia increases lung fluid and protein flow [33] producing edema. Thus, animals exposed to CNH should not develop pulmonary edema that could confound the observed weight stagnation during the CNH exposure.

Mean hematocrit was similar to arterial hematocrit values reported for rabbits [34–38] and increased to approximately 56% after 14 days of CNH. Similar hematocrit increases have been reported in adult New Zealand White rabbits after 7–10 days of hypoxia [36, 38]. On the other hand, blood glucose levels were similar between control and CNH rabbits and like those previously reported for healthy rabbits behaving or under light sedation [39]. Thus, rabbits exposed to CNH appear not to be subjected to a major stress in the hypoxic chamber. Moreover, because hyperglycemia reduces hypertension and modifies autonomic control in mice exposed to intermittent hypoxia [40], rabbits subjected to CNH have glycemic levels that should not interfere with responses to hypoxia.

Resting arterial blood pressure and heart rate were not significantly affected by the CNH exposure with respect to the control controls and were similar to those previously reported in rabbits [41–46]. Exposure of rats to 14 days of CNH does not affect the heart rate [8], and adult rats previously exposed to chronic hypobaric hypoxia (F_I_O_2_ = 0.12) for 3 weeks present the same heart rate as control animals in normoxia [20]. Thus, heart rate appears not to be significantly modified by either normobaric or hypobaric hypoxic challenges. Systolic, pulse and mean arterial pressure increase in adult rats exposed to hypobaric hypoxia (F_I_O_2_ = 0.12) during 10 days after birth, without modification of diastolic pressure or heart rate [32], although more moderate hypoxic levels (F_I_O_2_ = 0.14) appear to have no effects on these cardiovascular parameters [47]. Similarly, CNH increases both mean arterial blood and pulmonary pressure in rats [8]. Acute hypoxia increases arterial blood pressure and reduces rabbit heart rate linearly with blood oxygen saturation [43], but these changes appear not to modify the cardiovascular function of rabbits exposed to CNH.

In control animals, short-term (SD1) and long-term (SD2) HRV values were lower to those previously reported for awaken New Zealand White rabbits [42] although our values are consistent with previous reports that long-term variability was also greater than short-term variability [42]. This difference may arise from the cardiovascular and ventilatory effects of the anesthetic used in our experiments [48–50]. Rabbits exposed to CNH presented no significant changes in short-term variability but a significant reduction in long-term variability. A reduction in long-term variability without modification of short-term variability has been observed after heart isolation in rabbits [42]. Thus, this reduction of long-term variability observed in CNH rabbits may indicate a reduction in cardiac autonomic control. Accordingly, we used HRV spectrogram analysis as a surrogate of autonomic control to the heart, as previously described [42, 44, 51–54]. In the present study, we found that HRV spectrograms of control rabbits showed the largest peaks in frequencies below 0.2 Hz and above 0.8 Hz, with mean maximal around 0.06 Hz and 0.97 Hz, respectively. Similar frequency band distribution has been reported in anesthetized rabbits [55]. In our experiments, the changes in the higher frequency (> 0.8 Hz) components displayed by CNH rabbits appear not to be related to altered resting ventilation, since both control and CNH animals have similar ventilatory frequencies [56]. Exposure to CNH results in flattened HRV spectra, with a reduction of peak amplitude below 0.2 Hz, an almost complete absence of peaks above 0.8 Hz and a concomitant increase of the 0.2–0.8 Hz band power. The mean HRV spectrum of CNH-rabbits shows a large and significant reduction in the power of the highest and lowest peak frequencies of the studied range, with a significant increase in the power in the 0.2–0.5 Hz range, with no changes in the total power content of the spectrum with respect to control animals. It has been shown that parasympathetic blockade reduces the power content of the higher frequency spectral components (up to 0.6 Hz), while sympathetic blockade reduces the power of the lower part of the spectrum (up to 0.44 Hz) in unrestrained awaken rabbits [51]. Therefore, modification of the mean spectra observed in CNH rabbits suggests an important change in the balance of sympathetic-parasympathetic activity, giving rise to a new set-point in autonomic heart control.

### Limitations of the study

All the physiological recordings performed in our study were made under anesthesia. It has been previously shown that mostly all anesthetic agents normally depress cardiorespiratory function. Then, it is plausible that our results may underestimate the cardiovascular consequences of CNH exposure. However, it is worth noting that for both control and CNH-exposed animals, special care was taken when using anesthesia to have both experimental groups under the same anesthesia depth (Stage III plane 2). Therefore, both groups were comparable and even in the presence of anesthesia we observed an effect of CNH exposure on cardiovascular regulation. Nevertheless, we believe that future studies should address the consequences of CNH exposure on freely moving awake rabbits. Another limitation is that we did not directly measure sympathetic or parasympathetic discharges to the heart. We used HRV analysis as an indirect method to assess cardiac autonomic function. However, we performed bilateral vagotomy to assess the contribution of vagal modulation on spectral HRV bands. New and future studies should completely unveil the effects of CNH on cardiac autonomic drive by using selective pharmacological blockage of cardiac sympathetic/parasympathetic activity.

## Conclusions

Exposure to CNH produced no significant changes in cardiovascular variables but modified HRV. The HRV analysis showed that exposure to CNH is associated with increased long-term variability and a decrease in higher frequency components of the power spectra, suggesting a cardiac autonomic imbalance. Additionally, acute vagotomy further increase the HRV shift towards lower frequencies and larger power content strengthening the notion of autonomic imbalance.

## Abbreviations

CNH: chronic normobaric hypoxia
ECG: electrocardiogram
F_I_O_2_: oxygen inspiratory fraction
HF: high-frequency HRV band
HRV: heart rate variability
LF: low-frequency HRV band
PaO_2_: arterial oxygen partial pressure
PaCO_2_: arterial carbon dioxide partial pressure
PB: barometric pressure
PSD: power spectral density
SD1: short-term HRV
SD2: long-term HRV
VLF: very-low frequency HRV band
